# Life history, systematics and flight ability of the Early Permian stem-mayflies in the genus *Misthodotes* Sellards, 1909 (Insecta, Ephemerida, Permoplectoptera)

**DOI:** 10.1186/s12862-021-01820-x

**Published:** 2021-05-24

**Authors:** Pavel Sroka, Roman J. Godunko, Nina D. Sinitshenkova, Jakub Prokop

**Affiliations:** 1 Biology Centre of the Czech Academy of Sciences, Institute of Entomology, Branišovská 31, České Budějovice, CZ 370 05 Czech Republic; 2 Department of Invertebrate Zoology and Hydrobiology, University of Łódź, Banacha 12/16, 90237 Łódź, Poland; 3 State Museum of Natural History NASU, Teatralna 18, Lviv, Ukraine; 4 Palaeontological Institute of the Russian Academy of Sciences, Profsoyuznaya 123, Moscow, Russia; 5 Department of Zoology, Faculty of Science, Charles University, Viničná 7, Praha 2, CZ 128 00 Czech Republic

**Keywords:** Ephemerida, Misthodotidae, Functional morphology, Thorax, Sexual dimorphism, Mating behavior, Early Permian, Russia

## Abstract

**Background:**

The stem-group of Ephemeroptera is phylogenetically important for understanding key steps in evolutionary history of early pterygote insects. However, these taxa have been mostly studied from the taxonomy point of view focused on the pattern of wing venation and often using only classical optical microscopy devices. In-depth studies on detailed morphology of the different body structures are scarcely performed, although the results are critical for elucidation of life history traits and their evolutionary pattern among the basal pterygotes.

**Results:**

New information is presented on the morphology of two species of *Misthodotes*, which are stem-mayflies from the Early Permian. Based on new results obtained from a re-examination of the type specimens and supplementary material, we infer the life history traits of both the adult and larval stages of these Palaeozoic insects and reconsider previous interpretations. For the first time, we report the structure of the thoracic pleura and the articulation at the base of the wing in a stem-group of Ephemeroptera and compare them with those of extant mayflies. We also provide additional support for the systematic placement of investigated taxa and an amended diagnosis of the genus *Misthodotes*.

**Conclusions:**

Adult *Misthodotes sharovi* and *Misthodotes zalesskyi* had chewing mouthparts, which enabled them to scavenge or feed on plants. The wing apparatus was adapted for slow powered flapping flight and gliding, using long caudal filaments for steering. The wing base does not have rows of articulary sclerites as previously hypothesized for some Palaeozoic taxa but inflexible axilla similar to that found in modern mayflies. The structure of the thoracic pleura is also similar to that in the crown group of Ephemeroptera, while differences in the course of sutures may be explained by an evolutionary trend towards more powerful dorsoventral flying musculature and forewing-based flight (anteromotorism) in modern taxa. There is no evidence for swarming behaviour and mating in the air as occurs in modern mayflies as they had none of the associated morphological adaptations. Putative larvae of *Misthodotes* can not be unambiguously associated with the adults. They also exhibit some morphological specializations of Protereismatidae like 9 pairs of abdominal tracheal gills supporting their benthic lifestyle with legs adapted to burrowing.

**Supplementary Information:**

The online version contains supplementary material available at 10.1186/s12862-021-01820-x.

## Background

The Permoplectoptera are the Palaeozoic stem group of modern mayflies (Ephemeroptera) and one of the most ancestral lineages within the pterygote insects, and as such, they are very important for understanding the early steps in the evolution of this speciose group of organisms. The Permoplectoptera forms a sister group to Heptabranchia, which consists of the stem group Coxoplectoptera (Lower Cretaceous) and the crown group Ephemeroptera [46].

Very little is known about the life history of these insects, since most taxa are only preserved as isolated wings or wing fragments [8, 22]. Within the four families currently recognized in the Permoplectoptera, the family Misthodotidae is exceptional in that various parts of their bodies are well-preserved along with the wings in several specimens, allowing the study of complex body structures. Nevertheless, the available studies on Misthodotidae (mostly published several decades ago) are mainly concerned with taxonomy and mostly focus on the pattern of wing venation [8, 9, 13, 22, 43–45, 49]. Most species of Misthodotidae are described in the genus *Misthodotes*, which contains 11 species (5 from North America, 5 from Russia and one from Germany). Of all these fossils, 7 species are known only based on their wings or wing fragments. Kinzelbach and Lutz [22] provide a key to *Misthodotes* species, which includes the 8 species known at the time. Individual species may be distinguished mainly based on wing venation and wing length. Willmann [54] transferred some species of *Misthodotes* into the newly erected genera *Arnulfias* Willmann [54] and *Eurekter*  Willmann [54], mostly based on the shape of distal portion of veins C and Sc. This classification is not followed here, since our observations do not support these generic changes.

One of the richest sources of material of *Misthodotes* was found at the Early Permian locality of Tshekarda near the Sylva River in Russia (Perm Region), where two species co-existed, described as *Misthodotes sharovi* Tshernova [49] and *Misthodotes zalesskyi* Tshernova [49]. For both of them, the type series contain several nearly complete specimens with reasonable to excellent preservation. In addition to the adult specimens, alleged larvae of *M. sharovi* are described from the same locality. We reinvestigated all available material including holotypes of these two species of *Misthodotes* from the Permian of Russia and used novel ways of enhancing the knowledge of this evolutionary important taxon and reconstructing the life histories of both adults and larvae.

Specifically, in adults we aim to (i) reconstruct the flight characteristics based on the morphology of the wings, thorax and wing articulation, (ii) clarify the feeding habits based on the morphology of the mouthparts and associated structures, (iii) assess the mating behaviour and compare the morphological structures they share with modern mayflies. For larvae, we focus on (i) verification of their association with adult specimens and (ii) interpretation of their functionally significant morphological characters, such as legs, tracheal gills and caudal filaments in order to clarify the life history of the larva in the aquatic environment during the Permian period.

## Methods

We studied the material of *M. sharovi* and *M. zalesskyi* in the Palaeontological Institute of the Russian Academy of Sciences in Moscow, Russia (PIN hereafter). All specimens represent compression fossils. For several specimens, part and counterpart are available, marked as " + " and "—". The letters "a", "b", etc. refer to the presence of more specimens on the same slab with a unique number. Tshernova [49] studied 15 specimens of *M. sharovi* and 5 specimens of *M. zalesskyi* when describing those species. However, she did not explicitly mention any specimen except of the holotype to represent a part of the type series. Therefore, in compliance with ICZN articles 72.4–72.5 and 73 we do not treat these specimens as paratypes here. From this material, we selected specimens with a reasonable preservation of at least some body structures for a detailed study. Specimens consisting solely of wing fragments were not investigated in detail. The material studied includes the following specimens: *M. sharovi* adults: 1700/3209 (holotype, female), 212/26 (female), 1700/375 + and—(male), 1700/385 + and—(sex unknown), 1700/386 + and—(sex unknown), 1700/387 (male), 1700/388 + (male), 1700/392 (male), 1700/393 (sex unknown), 1700/393a (sex unknown), 1700/3211 + and—(sex unknown). *M. sharovi* larvae: 1700/374 + and—, 1700/379 + and—. *M. zalesskyi* adults: 1700/371 + and—(sex unknown), 1700/391 + and—(sex unknown). *Misthodotes* sp.: 1700/371a + and—(sex unknown), 1700/371b + and—(sex unknown).

### Locality and geological setting

The famous Tshekarda locality near the Sylva River, Perm District, is one of the exceptional uppermost Lower Permian insect localities with more than 8000 recorded specimens attributed to 25 orders and 99 insect families [1]. Fossils are dated to the Kungurian (279–272 Mya) and are preserved in the deposits in the Koshelevka Formation. The specimens examined were gathered during expeditions headed by A.G. Sharov from 1959 to 1961 (prefix 212, 1700) and collected by E.V. Permiakova and Z.I. Dzyu in various years [2]. Details of the locality can be found in Ponomaryova et al. [35], Aristov [2] and Aristov and Rasnitsyn [1]. The results of previous studies on megasecopterans reveal exceptional preservation of fine surface microstructures on some specimens and their suitability for study using environmental electron scanning microscopy [34].

### Optical devices and measurements

The material was examined dry and under a film of ethyl alcohol using stereomicroscopes Olympus SZX7, Leica M205 C and Nikon SMZ745T. The photographs of specimens examined dry or under a film of ethyl alcohol were taken using a Canon EOS 550D digital camera equipped with MP-E 65 mm and EF-S 60 mm macro-lenses. Original photographs were processed using image-editing software and some were processed by the stacking software Helicon Focus Pro (Helicon Soft, Kharkiv, Ukraine) or Zerene Stacker (Zerene systems LLC, Richland, U.S.A.). Photographs were sharpened and the contrast and tonality adjusted using Adobe Photoshop™ version CS6 (Adobe Systems Incorporated, San Jose, U.S.A.). Scanning electron micrographs were taken using an environmental electron microscope Hitachi S-3700 N (Hitachi Ltd, Chiyoda, Tokyo, Japan) at an accelerating voltage of 15 kV with a turntable sample holder at the National Museum in Prague. The measurements of individual body parts were taken either by using an ocular grid, or inferred from the photographs taken with a calibration scale.

## Results

The descriptions of *M. sharovi* and *M. zalesskyi* complement those of Tshernova [49], emphasizing the characters important for functional morphology, and those that are not specified in the original descriptions. Based on our observations, we also provide an amended diagnosis of the genus, complementing our previous knowledge on the species from North America.

**Imago of** ***M. sharovi*** **and** ***M. zalesskyi***

**Fig. 1:**
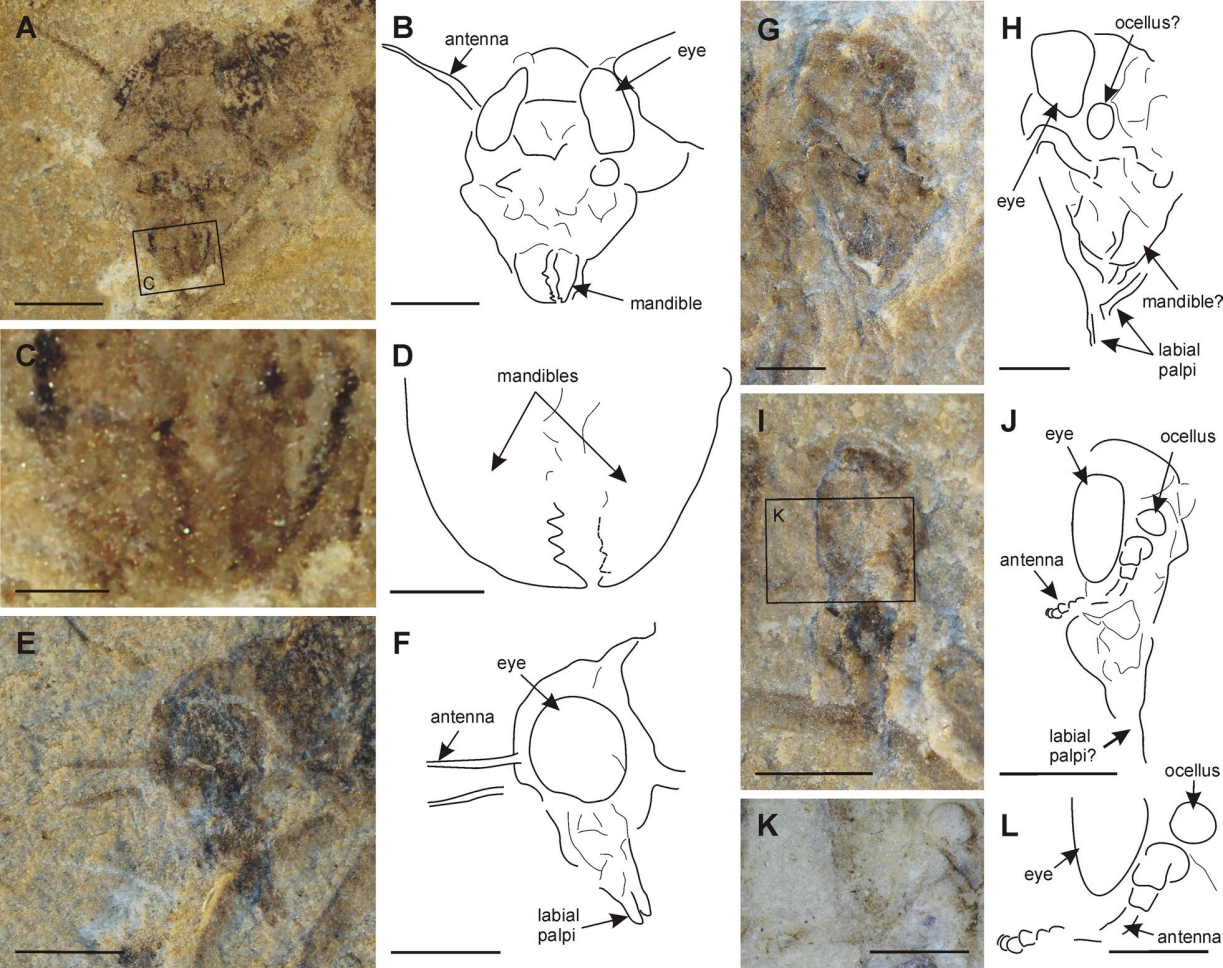
*Misthodotes* spp., head. **A**–**B** *M. sharovi* specimen 1700/3209, head; **C**–**D** *M. sharovi* specimen 1700/3209, detail of mandibles; **E–F** *M. sharovi* specimen 1700/388, head; **G-H** *M. zalesskyi* specimen 1700/371, head; **I**–**J** *M. sharovi* specimen 212/26, head; **K**–**L** *M. sharovi* specimen 212/26, detail of antenna (all photographs of dry specimens except K, which is viewed under a layer of ethanol; rectangles in figures A and I mark positions of the detailed figures **C** and **K**, respectively; scale bars represent **A**, **B**, **E**–**J** = 1 mm; **C**, **D** = 0.2 mm; **K**, **L** = 0.5 mm)

**Fig. 2:**
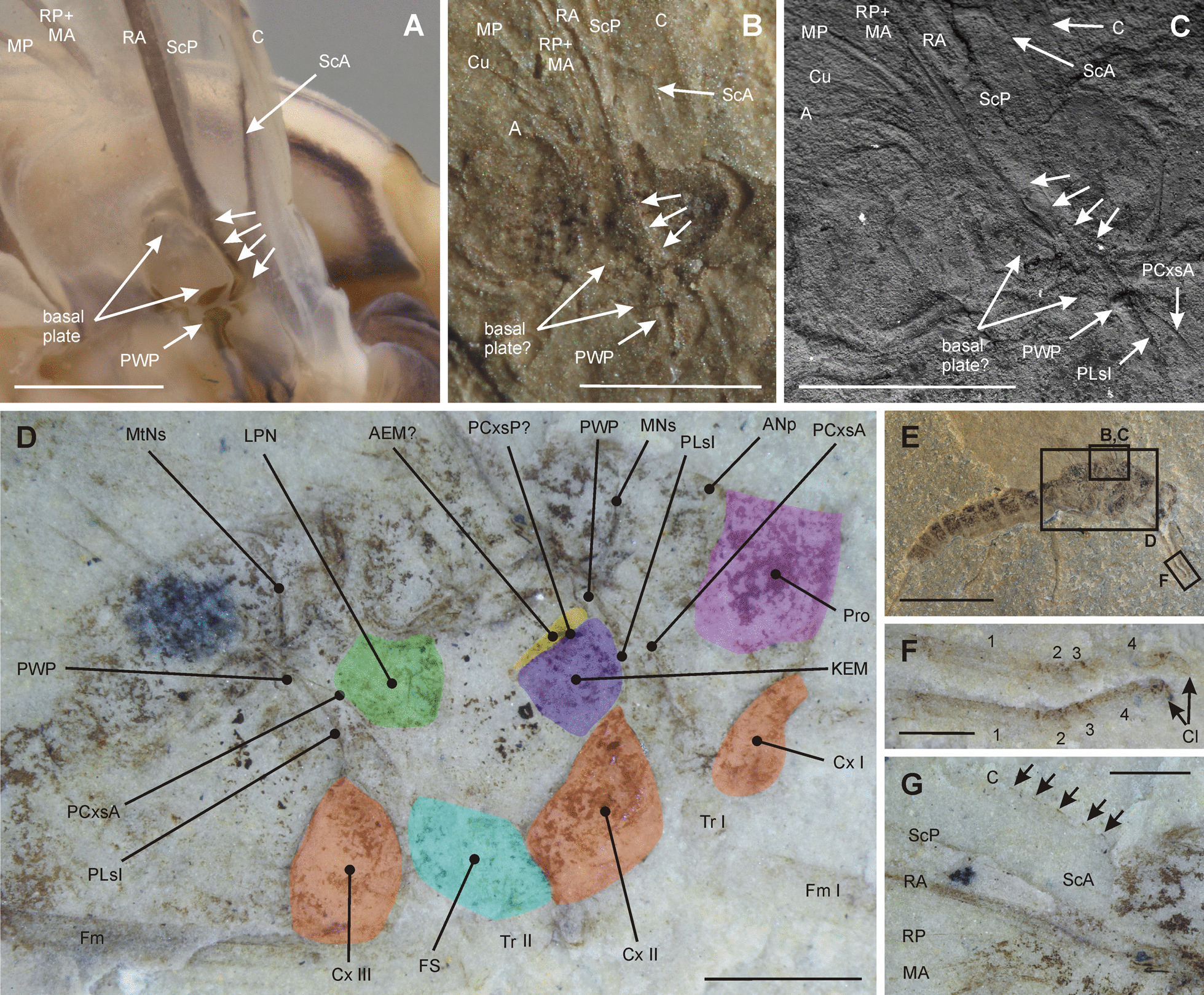
Wing base, thoracic pleura and legs of *M. sharovi* and recent mayflies. **A** *Siphluriscus chinensis*, lateral view of forewing base; **B** *M. sharovi* specimen 1700/386 + , lateral view of forewing base, photograph of dry specimen under oblique lighting; **C** *M. sharovi* specimen 212/26, lateral view of forewing base, photograph from ESEM; **D** *M. sharovi* specimen 212/26, lateral view of thorax, photograph under layer of ethanol; **E** body of *M. sharovi* specimen 212/26, with positions of detailed figures marked, photograph of dry specimen under oblique lighting; **F** *M. sharovi* specimen 212/26, foretarsi, photograph under layer of ethanol, numbers mark tarsal segments; **G** *M. sharovi* specimen 212/26, forewing base with arrows indicating spines on costa, photograph under layer of ethanol (in A-C, unmarked arrows point to basal curved part of radius adjacent to basal plate; scale bars represent **A**–**D** = 1 mm; **E** = 5 mm; **F**–**G** = 0.5 mm)

**Fig. 3:**
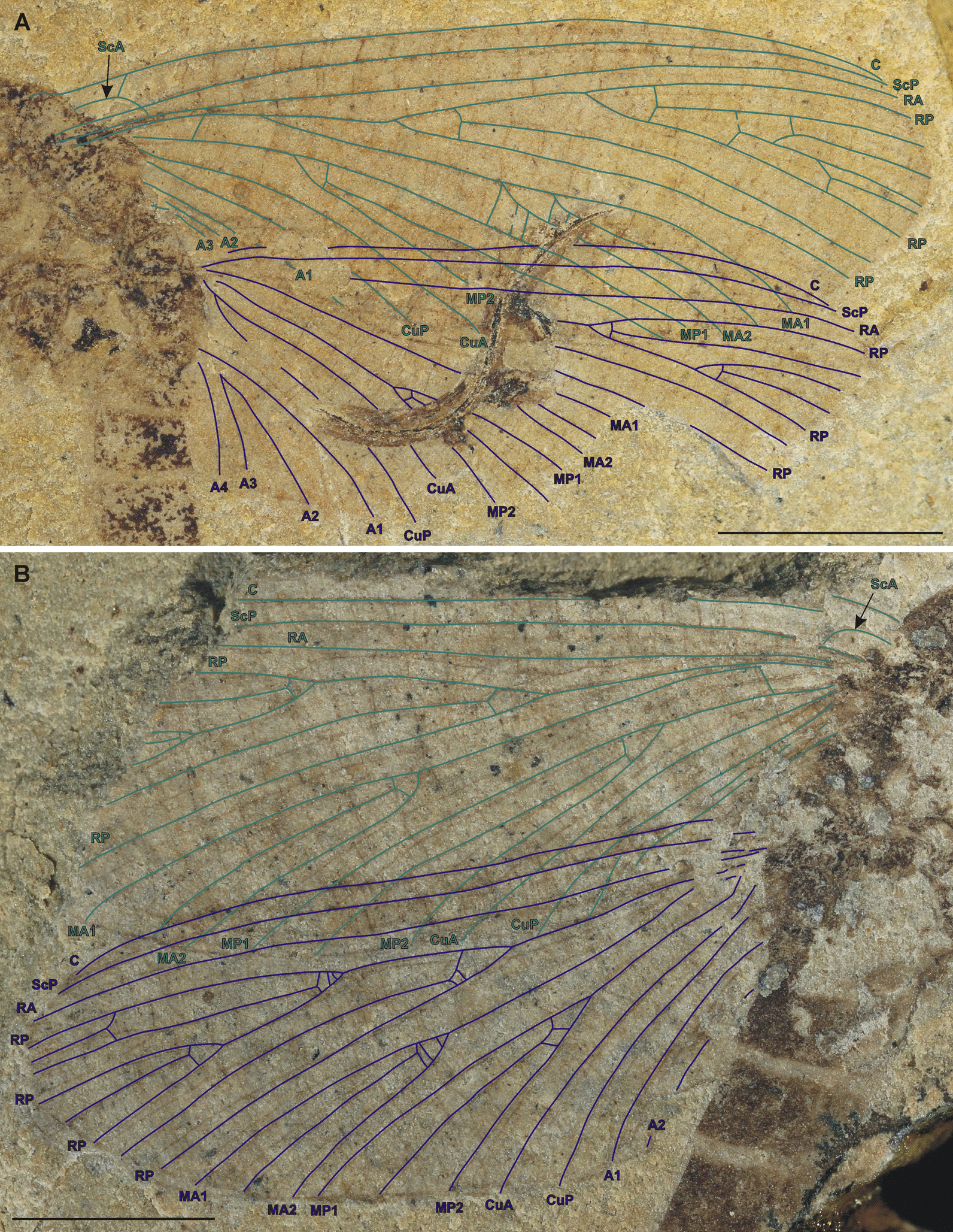
*Misthodotes* spp., wing venation. **A** *M. sharovi* specimen 1700/3209, photograph of dry specimen; **B** *M. zalesskyi* specimen 1700/391, photograph of dry specimen (scale bars represent 3 mm). Most cross veins not depicted

**Fig. 4:**
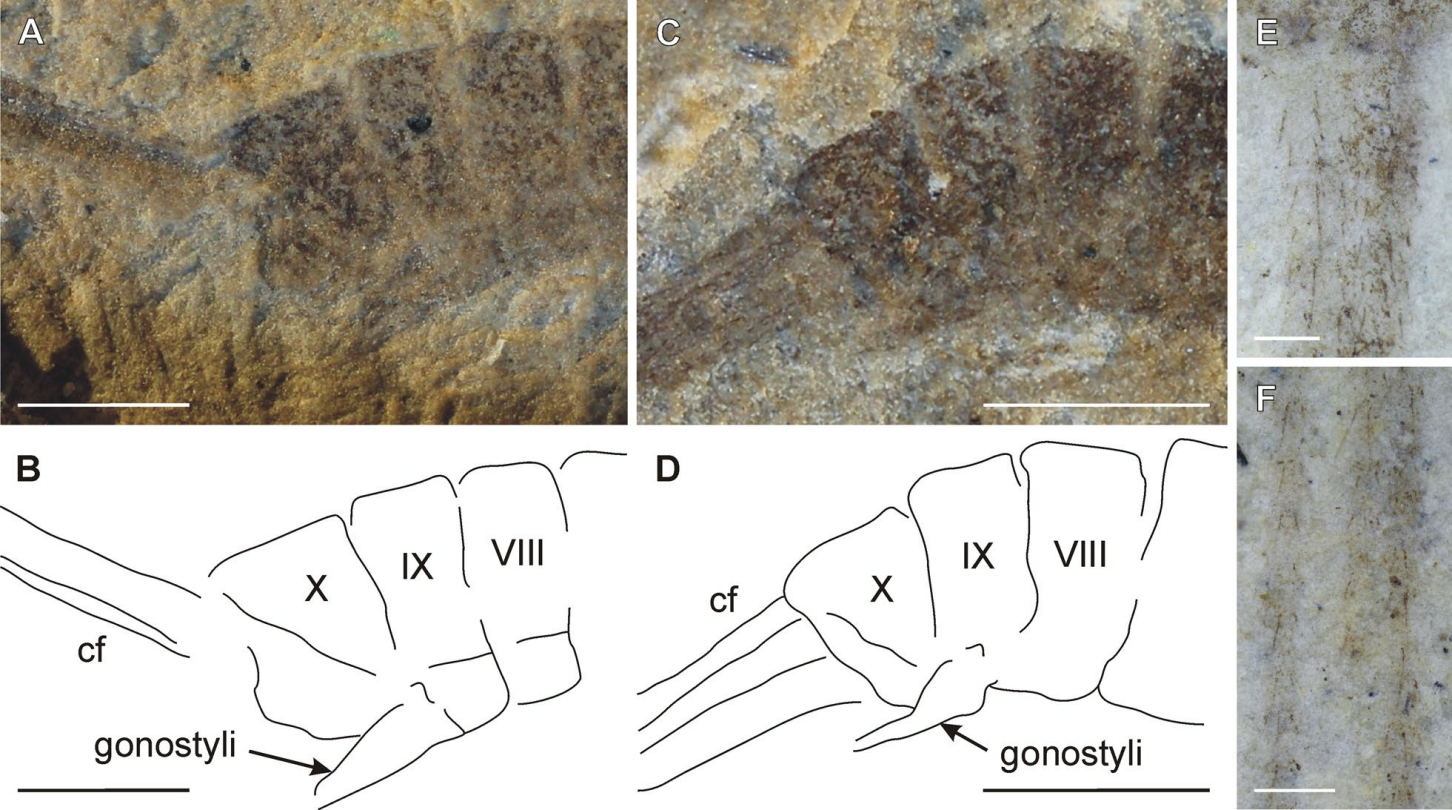
*Misthodotes* spp., abdomen. **A** *M. sharovi* specimen 1700/375—, distal part of abdomen, photograph of dry specimen; **B** the same, line drawing; **C** *M. sharovi* specimen 1700/392, distal part of abdomen, photograph of dry specimen; **D** the same, line drawing; **E–F** *M. sharovi* specimen 1700/388, details of setae on caudal filaments, photographs under layer of ethanol (scale bars represent A-D = 1 mm; E, F = 0.2 mm)

**(Figs.** 1, 2, 3, 4,** Additional file** 1:** Fig. S1**)

Since both species are very similar (see Discussion), we describe individual body structures together with remarks on what is discernible on which specimen.

***Summary measurements***: *M. sharovi* body length 9.67–15.33 mm, forewing length 8.30–12.63 mm, hind wing length 6.72–10.63 mm; *M. zalesskyi* body length unknown (body not entirely preserved), forewing length 12.88–15.00 mm, hind wing length 11.00–12.63 mm. For all measurements see Additional file 3: Table S1.

***Head***: in all specimens with a preserved head, it is apparent that the head capsule is orthognathous and elongated ventrally (1700/3209, 1700/388, 1700/3211, 1700/387, 1700/375, 1700/385, 1700/392, 212/26, 1700/371, 1700/371a). Anterior tentorial pits are discernible on specimens 1700/371 (Fig. 1G, H) and 1700/3209 (Fig. 1A, B). Compound eyes oval in shape, relatively large, proportions vary between specimens (Fig. 1). In all specimens the compound eyes do not appear to be divided into a lower and upper part (?1700/388, 1700/392, 1700/371, 1700/3209). Ocelli recognizable in 212/26 (Fig. 1I-L). Antennae partially preserved in four specimens, always incomplete, thin, filiform, with no recognizable setation (1700/3209, 1700/388, 212/26, 1700/375). Antennal bases situated behind the compound eyes (1700/3209, 1700/375, 1700/392). Antennomeres with scape and pedicel distinctly larger, flagellum with elongated flagellomeres, approximately 2 × longer than wide (1700/3209, 212/26). Paired mandibles with ?4 pointed teeth (apical tooth largest) on inner margin, molar area not visible (1700/3209, Fig. 1C, D). Elongated paired structures that protrude distally on head capsule are most likely labial palpi, although their segmentation is not clearly discernible (212/26, 1700/375, 1700/388, 1700/3209, 1700/3211, 1700/371, 1700/371a, Fig. 1E–J).

***Thorax***: prothorax the smallest thoracic segment, with distinctly sclerotized pronotum (Fig. 2D). Both wing-bearing segments, meso- and metathorax, are large and similar in size and general arrangement. Individual sclerites well visible in lateral view on several specimens (212/26, 1700/3209, 1700/371). Fine structure of pleura without visible setae or scales observable on specimens 212/26 and 1700/3209. Pleural wing process (PWP)^1^ is prominent and identifiable on both the meso- and metathorax (Fig. 2B–D). Near PWP there are two sutures on both the meso- and metathorax (Fig. 2C–D), namely the inferior pleural suture (PLsI) and anterior paracoxal suture (PCxsA). PLsI runs to the coxa and diverges from PCxsA over its entire length. Basal plate (BP) connecting PWP indistinct, basalare and subalare not recognizable (Fig. 2B, C). Indistinct anteronotal protuberance (ANp) on mesonotum. Transversal mesonotal suture (MNs) clearly discernible. Analogous suture visible also on metathorax. Sclerotized area posterior to PLsI probably is a katepimeron (KEM). Presence of posterior paracoxal suture (PCxsP) dividing KEM from anepimeron (AEM) is not distinct. Lateropostnotum (LPN) recognizable posteriorly and furcasternum (FS) ventrally on mesothorax.

Both pairs of wings are very similar in size and arrangement. Forewings 1.2–1.3 × longer than hindwings. Both fore- and hind wings are oval, with a length/width ratio of 2.7–3.1:1 for the forewing and 2.5–3:1 for the hind wing. Venation is very similar on both wings (Fig. 3). Both pairs of wings with hyaline membrane without any maculations. Costal brace present, arched, not touching C, distally connected to RA, (Figs. 2B, C; 3). One oblique cross vein present between C and costal brace. Row of regularly arranged spines discernible on basal part of the costa in specimen 212/26 (Fig. 2G). Vein CuA simple and thus lacking the triad. Anal brace not clearly discernible on available specimens. Microtrichia along posterior wing margin not recognizable, apparently absent. Cross veins numerous, straight or slightly oblique and regularly spaced (Fig. 3), but clearly less numerous than in representatives of Protereismatidae.

Legs preserved to some extent in almost all specimens, but only rarely are all segments discernible (see Additional file 3: Table S1). Patella with suture not clearly discernible (possibly present on meso- and metathoracic leg in 1700/392), tibia and tarsus with longitudinal rows of short setae (Fig. 2F). Length ratio of femur:tibia:tarsus 1:0.9:1 for forelegs (mean values, measurable in three specimens), 1:0.8:? middle legs (measurable in one specimen, tarsus not preserved), 1:1:0.6 for hind legs (measurable in one specimen); for all measurements see Additional file 3: Table S1. Tarsi with four tarsomeres, second and third tarsomere of same length and approximately 0.4 × length of the first tarsomere and 0.3 × length of the fourth tarsomere (Fig. 2F). Pretarsus with double claws.

***Abdomen***: elongated, consisting of ten visible segments and caudal appendages of segment eleven. Terga darker than sterna. Structural details of neither male nor female genitalia preserved in any specimen, male genitalia only faintly visible in specimens 1700/392 and 1700/375, discernible by the gonocoxal lobes and claspers (multijointed gonostyli) on segment IX (Fig. 4A–D). Caudal filaments at least partially preserved in specimens 1700/388, 212/26, 1700/392 and 1700/387. Cerci very similar to paracercus, of same thickness and covered with short spine-like setae (Fig. 4E, F). In 1700/388, two filaments are completely preserved, 1.8 × longer than body.

**Larva of** ***M. sharovi***

(**Figs.** 5, 6)

**Fig. 5:**
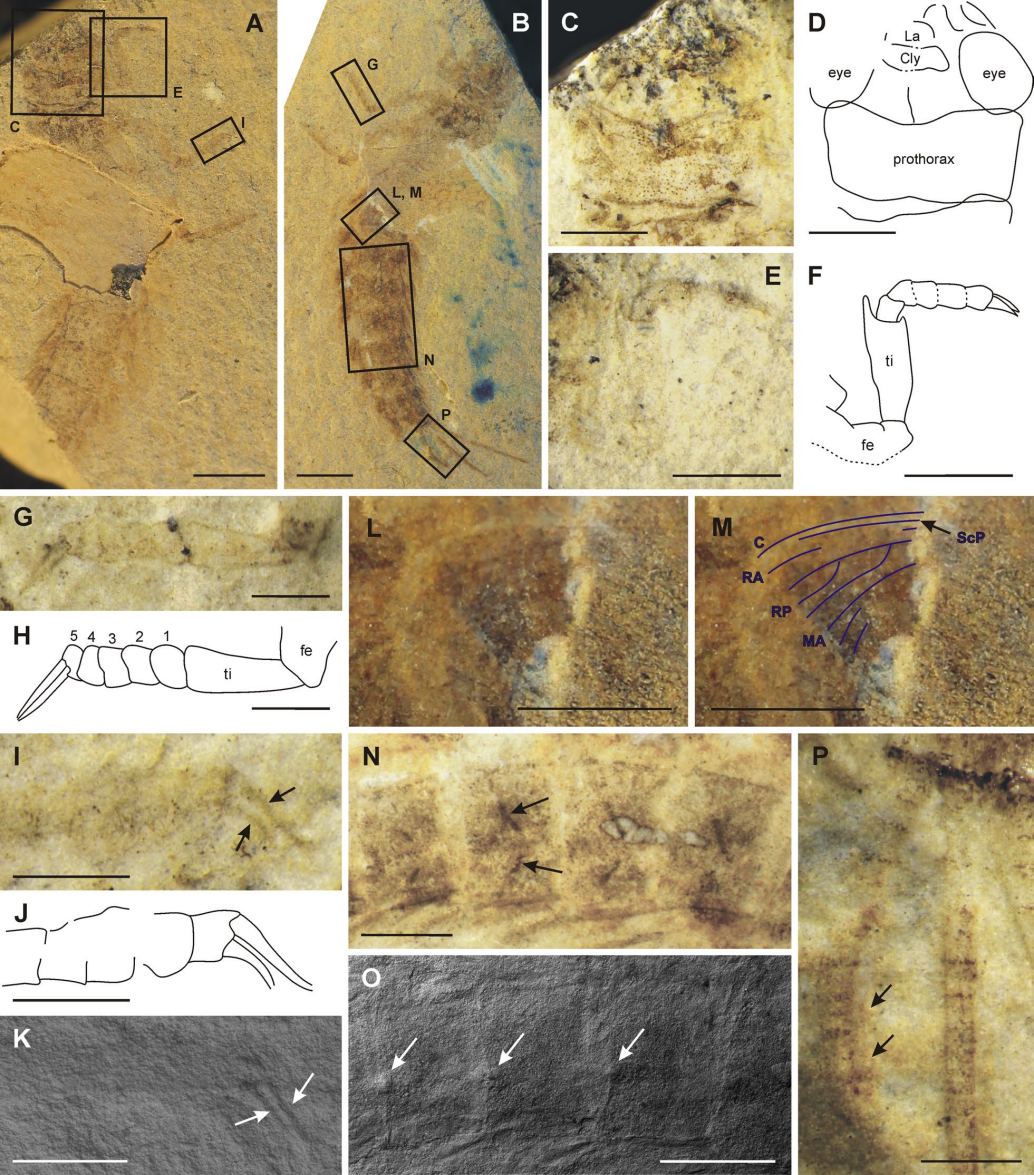
*M. sharovi*, larva. **A** specimen 1700/379 + ; **B** specimen 1700/379—; **C**, **D** head and prothorax; **E**, **F** foreleg; **G**, **H** distal portion of middle leg, numbers mark tarsal segments; **I**–**K** detail of middle tarsus, arrows mark tarsal claws; **L** hind wing pad; **M** reconstruction of vein precursors; **N** middle portion of abdomen, arrows mark dark oblique stripes; **O** middle portion of abdomen, arrows mark projections on terga; **P** caudal filaments, arrows mark traces of swimming setae (photographs **A**, **B**, **L**, **M** of dry specimen, **C**, **E**, **G**, **I**, **N**, **P** under layer of ethanol, **K**, **O** from ESEM; rectangles in figures A and B mark positions of detailed figures; scale bars represent **A**, **B** = 2 mm; **C**–**F**, **L**–**O** = 1 mm; **G**–**K, P** = 0.5 mm)

**Fig. 6:**
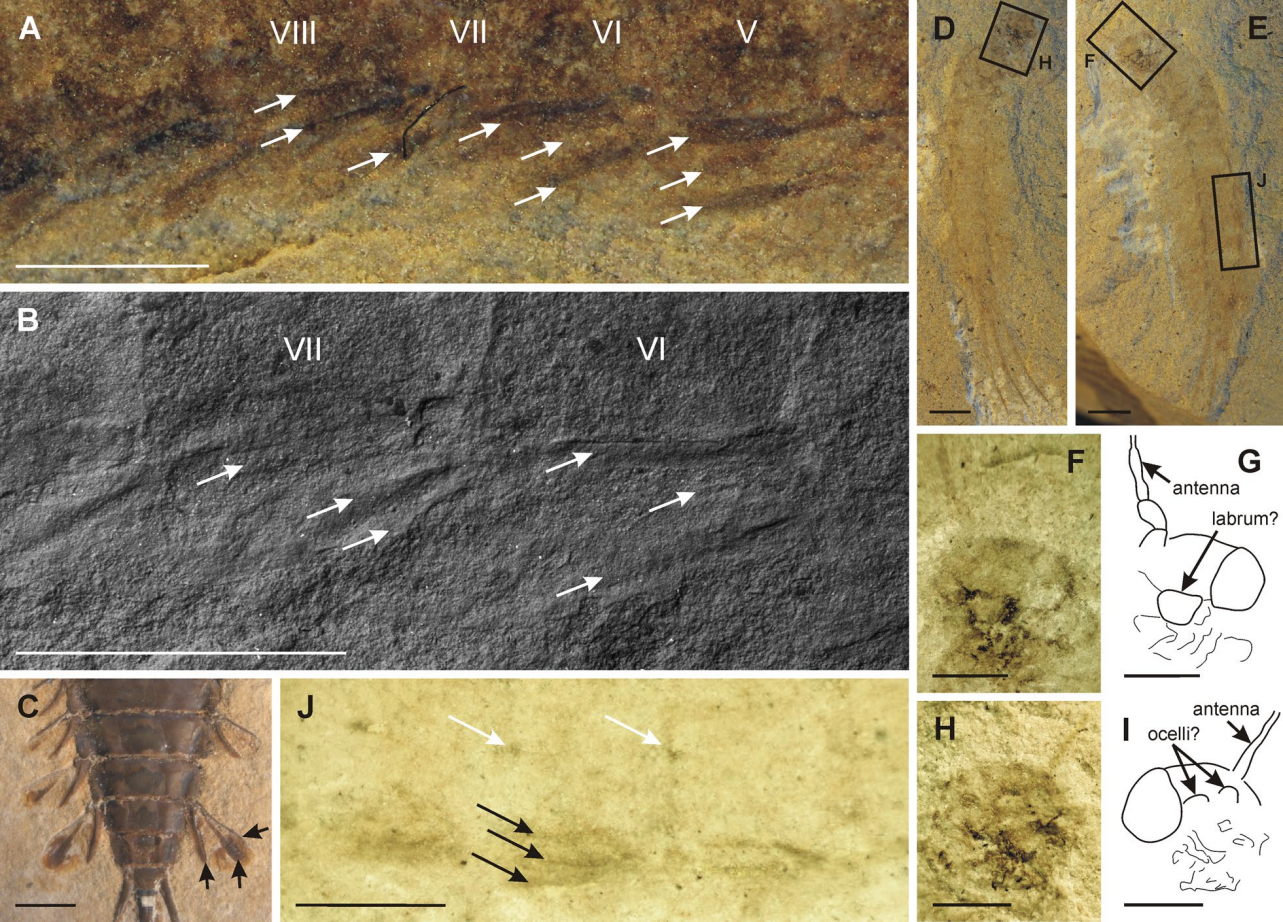
*M. sharovi* larva and comparative material of Hexagenitidae. **A** *M. sharovi* specimen 1700/379—, gills, arrows mark sclerotized ribs; **B** *M. sharovi* specimen 1700/379—, detail of gills under ESEM, arrows mark sclerotized ribs; **C** *Cratohexagenites minor* Staniczek, 2007, holotype, distal portion of abdomen, arrows mark sclerotized ribs on gills; **D** *M. sharovi* specimen 1700/374 + ; **E**, *M. sharovi* specimen 1700/374—; **F**, **G** *M. sharovi* specimen 1700/374—, head; **H**, **I** *M. sharovi* specimen 1700/374 + , head; **J** *M. sharovi* specimen 1700/374—, middle portion of abdomen, black arrows mark sclerotized ribs on gills, white arrows mark projections on terga (photographs **A**, **C**, **D**, **E** of dry specimen, **F**, **H**, **J** under layer of ethanol, **B** from ESEM; rectangles in figures D and E mark positions of detailed figures; scale bars represent **A**, **B**, **F**–**J** = 1 mm; **C**, **D**, **E** = 2 mm)

***Summary measurements***: body length 14.6 mm, cerci and paracercus incompletely preserved. For all measurements see Additional file 3: Table S1.

Of the two specimens mentioned by Tshernova [49], specimen 1700/379 is the best preserved, whereas the second specimen (1700/374) is rather poorly preserved. We provide a complementary description of these two specimens separately.

Specimen 1700/379 is preserved in a dorsolateral position, body fusiform. Surface of body densely covered by tubercles approximately 70–100 µm in diameter (Fig. 5C). Part 1700/379 + (Fig. 5A) with distal portion of head missing along with part of legs, metathorax and most of mesothorax, lacking abdominal segments I–II and VII–X. Counterpart 1700/379— (Fig. 5B) almost complete, missing structures include antennae, distal part of head, all legs on one side of body, posterior part of mesothorax and metathorax, and distal portion of caudal filaments.

***Head*** (Fig. 5C, D) prognathous, slightly narrower than prothorax, without traces of keel and/or projections. Distal portion of head missing, at least one eye preserved and almost complete, more than twice as long as wide. Epicranial suture partially preserved, probably widely V-shaped distally. Clypeus relatively short, not expanded laterally. Labrum nearly rectangular, narrower than clypeus. Traces of mandibles visible, short and robust, slightly curved inwards. Denticulation on mandibles not preserved. Antennae not clearly distinguishable.

***Thorax***. Prothorax more than two times wider than long, slightly trapezoidal, lateral margins diverge anteriorly, with protruding anterolateral corners rounded apically (Fig. 5C, D). Mesothorax deformed, only partly preserved. Mesothorax approximately the same width as prothorax, only one forewing-pad is partly preserved, with no traces of venation. The division between meso- and metathorax is hardly distinguishable. However, metathorax seems to be slightly shorter than mesothorax. Distal part of hind wing pad is preserved (Fig. 5L, M), visible distal ends of precursors of radial veins with two presumable forks. Vein precursors in medial field markedly bent distally.

Legs on one side missing. Foreleg (Fig. 5E, F) considerably shorter than middle and hind legs (Fig. 5A, B). Foreleg poorly preserved, femur relatively broad, longer than wide, possibly with stout spines of different length along outer margin. Tibia longer than femur, distinctly enlarged distally, with expanded outer projection and at least two stout pointed spines apically. Tarsus of approximately same length as tibia, terminating in two tarsal claws, slightly bent distally. Tarsus with 5 tarsomeres, tarsomere V slightly enlarged distally, position of individual tarsomeres shifted due to fossilization. Middle and hind femora with subapical transverse row of short stout setae distally. Middle leg with relatively long femur, slightly bent centrally. Middle tibia wider apically, seems rather asymmetrical at tip. Middle tibia shorter than femur, tarsus shorter than tibia, 5 tarsomeres, all approximately equal in length (Fig. 5G, H). Two tarsal claws up to 0.5 × length of tarsus, relatively straight, probably sharply pointed apically (Fig. 5G–K). Hind leg with femur relatively slender and long, slightly bent proximally. Tibia short and slender, not widened distally. Tarsus markedly longer than tibia, with 5 tarsomeres of approximately equal length. Tarsal claws as long as approximately 0.5 × length of tarsus, not bent, sharply pointed apically.

***Abdomen.*** Of ten segments, terminates in three caudal filaments. Abdominal segments gradually narrow distally, segments IX and X are the shortest. Two oblique dark stripes on abdominal segments IV–X (Fig. 5N). Posterolateral angles of individual segments triangular and seem to be well chitinized. Terga with well sclerotized projection on posterior margin of segments III–IX centrally (Fig. 5O). Posterior margin of segments V–X with traces of row composed of stout triangular spines. Tracheal gills on segments I–IX, inserted posterolaterally. Each gill consists of a single plate-like lamella with no traces of filaments. Gills VIII–IX the smallest. Three more or less preserved sclerotized ribs visible at least on gills II–VIII (Fig. 6A, B), present also on gill IX, but this structure is poorly preserved). Two ribs running along inner and outer margin of gill plate, up to approximately the middle of the length of the lamella. Third rib positioned centrally. Caudal filaments composed of segments of various length which terminate apically by row of stout triangular spines. Traces of swimming setae along inner side of cerci on both sides of paracercus (Fig. 5P). Paracercus basally the same diameter as cerci, most probably of comparable length, although apical parts of caudal filaments not preserved.

Specimen 1700/374 is preserved in ventral position, body fusiform (Fig. 6D, E). The imprint is poorly preserved. Head is preserved with fragment of antenna, scape and pedicel most probably of equal length (Fig. 6F–I). Details of mouthparts hardly distinguishable, most probably a trapezoidal-like labrum anteriorly. Poorly preserved traces of eyes as long as approximately half of the length of the head; eyes widely spaced. Segmentation of thorax hardly visible, prothorax shorter than meso- and metathorax, meso- and metathorax approximately equal in length, no traces of wing pads. Preservation of legs fragmentary; part of foreleg (coxa, part of femur), middle coxa, hind coxa and part of hind femur recognizable. Terga with sclerotized projection on posterior margin of segments III–IX centrally (Fig. 6J). Tracheal gills on abdominal segments I–IX, inserted in posterolateral angles of respective segments. Gills consist of single plates, no traces of filament bundles, gill size gradually decreasing from gill I to IX. Gills VIII–IX smallest, relatively narrow. Two to three visible sclerotized ribs on gills I–IX, two ribs along inner and outer margin of gill, third rib medially, close to inner margin (Fig. 6J). Three relatively robust caudal filaments almost entirely preserved, except for the distal part; preserved part of paracercus of the same length as cerci; width of paracercus near base equal to that of cerci. The traces of a few swimming setae sparsely scattered along inner margin of cerci and on both sides of paracercus.

**Amended diagnosis of** ***Misthodotes*** **Sellards, 1909**

Based on our study of the material of *M. sharovi* and *M. zalesskyi*, we provide additional generic diagnostic characters to those presented by Carpenter [7, 8].

**Imago:**

(i) chewing mouthparts, dentate mandibles, protruding labial palpi; (ii) antennae filiform; (iii) forelegs shortest; (iv) tarsi with four tarsomeres, second and third tarsomere the shortest; (v) double tarsal claws; (vi) wings oval, nearly homonomous; (vii) costal margin serrated; (viii) costal brace present, separated from costal margin; (ix) CuA either simple or with short terminal fork, lacking triad; (x) crossveins numerous; (xi) abdomen long, cylindrical; (xii) cerci and paracercus very long.

**Larva:**

(i) body fusiform; (ii) head prognathous, clypeus not elongated and not expanded anteriorly; (iii) mandibles robust, relatively short; (iv) prothorax shorter than meso- and metathorax, with nearly rectangular posterior angles; (v) metathorax slightly shorter than mesothorax; (vi) forefemur relatively broad, possibly with stout spines along outer margin; (vii) transverse row of short stout setae distally on middle and hind femora and tibiae; (viii) tarsi of all legs of equal length or shorter than respective tibiae, 5-segmented, terminate in two relatively long, straight and apically pointed claws; (ix) abdominal segments gradually narrow distally, with posterolateral angles triangular, abdominal segments IX and X the shortest; (x) abdominal terga with sclerotized projection on posterior margin centrally; (xi) tracheal gills on segments I–IX, gills VIII–IX smallest; (xii) each gill consists of single plate-like lamella; (xiii) gills II–VIII with three sclerotized ribs (two ribs along gill margins, one central rib which can be situated also close to inner margin); (xiv) three caudal filaments with swimming setae typical of Ephemeroptera.

## Discussion

**Remarks on taxonomic status and type material of** ***M. sharovi*** **and** ***M. zalesskyi***

There are some inconsistencies between the original paper of Tshernova [49] and the type material regarding the labelling of individual specimens. In the list of material, Tshernova [49] mentions that *M. zalesskyi* specimen No. 1700/371 consists of positive and negative imprints. In fact, there are three specimens on this piece, visible in both imprints (Additional file 1: Fig. S1). Two of them are marked 1700/371 and 1700/371a, the third is not marked. Specimen 1700/371 undoubtedly is the *M. zalesskyi* of Tshernova [49]; the photograph of this specimen presented as *M. zalesskyi* in the original description being unambiguously identifiable (Tshernova [49], Table 1, Fig. 1).

Specimen number "1700/371a" is cited in the legend of the figure of the drawing of the head and anterior part of thorax of *M. zalesskyi* in Tshernova ([49], Fig. 1), but does not appear in the list of material of *M. zalesskyi* or anywhere else in the text. Specimen 1700/371a is very small (body length 7.7 mm), well outside the range given for *M. zalesskyi* by Tshernova [49] as 18–19 mm. Moreover, there is a foreleg depicted in Tshernova's Fig. 1, which is present only on 1700/371 of all three specimens on this piece. We thus consider that there is an error in the legend of this figure in Tshernova ([49], Fig. 1) and conclude that Tshernova did not include this specimen in her study, and Fig. 1 in Tshernova [49] actually depicts specimen 1700/371. The third specimen with no assigned number on the same piece along with 1700/371 and 1700/371a is not mentioned at all in Tshernova [49]. It is preserved in a different sedimentary layer from the other specimens, which indicates burial at another interval of time.

Based on our observations on 1700/371a and a third, unmarked specimen on the same slab (tentatively treated as 1700/371b), both of which have two pairs of nearly homonomous wings with a costal brace and venation congruent with *M. sharovi* and *M. zalesskyi*, and the elongated head capsule and morphology of thoracic pleura of 1700/371a and 1700/371b, which also correspond with *M. sharovi* and *M. zalesskyi,* we assume them to be conspecific with one of these species.

One more discrepancy concerns *M. sharovi*. The holotype has catalogue number 1700/3209. This is stated in the text of Tshernova [49] and physically marked on the specimen. Other numbers (1700/3290 and 1700/320) appear in the caption of Table 1, Fig. 2 and Fig. 5 in Tshernova [49], respectively. The above mentioned figure legends in Tshernova [49] most probably contain typing errors.

**Differentiation between** ***M. sharovi*** **and** ***M. zalesskyi***

In the original descriptions of both *M. sharovi* and *M. zalesskyi* [49], differences between these two co-occurring species are paradoxically not explicitly mentioned. Both species are compared separately only with the North American species *M. ovalis*, *M. obtusus* and *M. biguttatus*.

In the identification key for *Misthodotes* in Kinzelbach and Lutz [22], several diagnostic characters are used to separate eight species of *Misthodotes* (including *M. sharovi* and *M. zalesskyi*), all using only wing venation. When we focus on *M. sharovi* and *M zalesskyi*, the only character directly separating these two species in the key of Kinzelbach and Lutz [22] is the width of fields between Rs and R, and between Rs and MA, which should be wider in *M. sharovi* than in *M. zalesskyi*. However, even if we compare the drawings in Kinzelbach and Lutz [22] there is no significant difference between these two particular species (Fig. 1B, H in [22]). This is in accordance with our observation on the venation of the type material of both species, where we found no significant differences (compare Fig. 3A and B).

We presume that Tshernova separate these two species based on the larger size of *M. zalesskyi*. An entire body is not preserved in any of the specimens of *M. zalesskyi*, thus the body length given by Tshernova [49] of 18–19 mm is probably only assumed. Wing dimensions are more reliable, for which Tshernova [49] cites 14–18 mm for the forewing of *M. zalesskyi* and 9–12 for *M. sharovi*. According to our measurements (Additional file 1: Table S1) there is no marked difference in size of the forewings of *M. sharovi* and *M. zalesskyi* as the difference between the largest specimen of *M. sharovi* and the smallest specimen of *M. zalesskyi* is less than the variation in size within *M. sharovi* (Additional file 2: Fig. S2).

The size difference between these two species is complicated by the possible intraspecific variability in size between sexes and generations. In recent mayflies, females are usually larger than males. However, in most specimens of *M. sharovi* and all specimens of *M. zalesskyi*, sex can not be reliably identified (see discussion on genitalia and eye size below). In recent mayfly species with more than one generation per year, pronounced differences in size also exist between individual generations, typically with the summer generation much smaller than the spring generation (e.g. [11]). This might also have been the case for fossil mayfly-related taxa, such as *Misthodotes*. Therefore, we consider the differentiation of these two species based only on size as highly unreliable, especially when it is as highly variable, as in case of *M. sharovi* and *M. zalesskyi*. It is possible that there are more than two closely related species in the Tshekarda deposit. It is also possible that all the specimens belong to a single species and the difference in size are due to differences between sexes and generations. We conclude that in the absence of other morphological characters for distinguishing particular species, we prefer to retain the original attribution designated by Tshernova [49].

**The functional and systematic significance of the morphology of** ***Misthodotes*** **species from Tshekarda lagerstätte**

Here we discuss the morphological characters of both *M. sharovi* and *M. zalesskyi*, compare them with the original descriptions of Tshernova [49] and draw conclusions about the life history traits of these insects. We deal with the adult and larval stages separately and also discuss their association. To distinguish possible subimagos from imagos within our material of *M. sharovi* and *M. zalesskyi*, we checked for the presence of setae along the wing margins, which is one of the most distinct traits of a subimago [14, 15, 40]. We found no indication of such setae, despite good preservation of minute setae elsewhere on the body (Fig. 4E, F). Therefore, we consider all these specimens to be imagos.

**Imago**

**Eyes and visual orientation**

The compound eyes are described as "relatively small" in *M. zalesskyi* by Tshernova [49]. For *M. sharovi*, there is no information on the size of its eyes in the original description, although there are three figures depicting eyes (Figs. 3, 4, 5 in Tshernova [49]). In modern mayflies, there is usually a high level of sexual dimorphism in the size of their eyes. Males tend to have distinctly larger eyes, which enable them to visually locate females in a swarm [40]. Based on our observations it is highly likely that the eye size differed between sexes also in *M. sharovi* and *M. zalesskyi*. Relatively large compound eyes are distinguishable in *M. sharovi* specimens 1700/392 and 1700/388 (Fig. 1E, F), whereas comparatively smaller eyes are visible in the holotype of *M. sharovi* (1700/3209) and *M. zalesskyi* specimen 1700/371. The comparatively small eyes of the *M. sharovi* holotype (1700/3209), where no genitalia are visible resulted in Tshernova [49] identifying it as a female. The overall body size of 1700/3209 is the largest in the *M. sharovi* type series, which also supports it being a female. On the other hand, specimen 1700/392 with larger eyes is most likely a male, as although barely discernible, it has male genitalia with elongated gonostyli on segment IX (Fig. 4C, D). The sexually dimorphic eye size indicates visual location of females by males. Ocelli are also distinguishable on several specimens, although not mentioned by Tshernova [49], which are similar in size and position to those of recent mayflies (Fig. 1I–L).

**Mouthparts and feeding habits**

The maxillary palpi and contours of other unspecified mouthparts are identified in *M. sharovi* adults by Tshernova [49]. She claims that the mouthparts were functional and of the chewing type. In modern mayflies, adult mouthparts are always vestigial and non-functional [40]. In Protereismatidae, a permoplectopteran family closely related to Misthodotidae, Tillyard ([48], 118) reports that the adults have reduced mouthparts.

Based on our observations on *M. sharovi* and *M. zalesskyi*, for all the specimens with a preserved head the head capsule protrudes ventrally (Fig. 1). Apical portion of this protuberance sometimes consists of several rounded, elongated and paired lobe-like structures (Fig. 1E–J), possibly labial palps, although there is no trace of segmentation. Staniczek et al. [47] describe similar elongated three-segmented labial palpi in imago of *Mickoleitia longimanus* (Coxoplectoptera) from the Lower Cretaceous of the Crato formation in Brazil.

In the holotype of *M. sharovi*, a pair of large mandibles are clearly visible, which have acute teeth along their inner margins (Fig. 1A–D). We assume these mouthparts are for chewing, based on their shape, denticulation and position. Mandibles are also usually the most sclerotized structures and most likely to be preserved. Other specimens have similar paired mouthparts, but the preservation of the detail is poor (Fig. 1G, H). Based on the presence of chewing mandibles, their distinctly protruding head is not adapted for piercing and sucking as in coeval Palaeodictyopterida, where both the mandibles and maxillae are elongated into stylets [38]. The structure of the mouthparts also does not indicate any significant reduction in the size of the mouthparts, we thus concur with Tshernova [49] that they were fully functional and used for chewing. A protruding head capsule associated with chewing mouthparts is present in several insect orders (e.g., Mecoptera, Diptera and some Coleoptera) and in the Mecoptera enables them to insert their head deep inside the body cavity of invertebrates [33]. In other taxa it can also serve the same purpose when feeding deep inside plant tissue. We did not record any other predatory adaptations in *M. sharovi* and *M. zalesskyi*, like raptorial forelegs or legs equipped with long spines for catching prey in flight as in contemporary damselflies [12]. We thus conclude they were probably either scavengers of other invertebrates or plant-feeders.

**Thorax and flight ability**

In *M. sharovi* and *M. zalesskyi*, the size and general arrangement of both the meso- and metathorax is very similar. In contemporary mayflies, the mesothorax is much larger than the metathorax, as a result of the evolutionary shift from the homonomous wings of Permoplectoptera to forewing-based flight (anteromotorism) and reduction in hind wings in modern Ephemeroptera. The reduction in the metathorax of mayflies is an apomorphic condition compared to other pterygote insects [51]. This is also in accord with the situation in the Late Carboniferous representatives of the stem group of Ephemerida, such as the Syntonopteroidea, whose hindwings were even markedly larger than the forewings, but their thoracic morphology is poorly documented [36].

***Pleural sclerites***

Tshernova [49] does not mention the structure of the pleura in *M. sharovi* and *M. zalesskyi*. Since details of their structure are observable in several specimens, we provide a description of individual components of this region of the thorax. If we compare the arrangement of the mesothoracic pleura in *M. sharovi* and *M. zalesskyi* as representatives of stem-mayflies with modern Ephemeroptera, it is possible to draw several conclusions. In both groups, there is a prominent pleural suture. In mayflies, this suture runs from the pleural wing process (superior pleural suture sensu Kluge [26] and divides into the prominent anterior paracoxal suture and inferior pleural suture (which continues towards the coxa) and posterior paracoxal suture. In Neoptera, the basic scheme is similar, although the course of the sutures differs slightly and the anterior paracoxal suture is much less developed than in mayflies. In *M. sharovi* and *M. zalesskyi*, superior pleural suture and inferior pleural suture are separate near the pleural wing process and neither is prominent (Fig. 2C, D).

The strengthening of the anterior paracoxal suture and its partial fusion with the inferior pleural suture in crown group mayflies serves to prevent the pleuron from distorting during active flight, due the strain associated with the enlargement of the dorsoventral muscles, particularly the scuto-episternal muscle [25]. According to Brodsky [5], enlargement of the tergosternal muscles in modern mayflies is closely associated with the evolution of swarming behaviour, for which it is necessay to be able to fly in a swarm close to many other individuals. We hypothesize that the fusion and strengthening of the pleural suture in crown Ephemeroptera was connected with a change in mating behaviour from individual encounters to mass swarming. Thus, it is possible that mating in *M. sharovi* and *M. zalesskyi* was dependent on individual encounters. This assumption is also consistent with the presence of functional mouthparts, since mass swarming occurs frequently in insect taxa with non-functional mouthparts and a very short adult lifespan, including all the contemporary mayflies [41]. It is also supported by the morphology of the legs (see below).

In the phylogeny of the mayfly lineage, mass swarming and associated morphological structures are probably an apomorphy of crown group Ephemeroptera. The pleural region is not preserved sufficiently in Coxoplectoptera for drawing conclusions about the fusion and strengthening of the pleural suture in this group (see [47]: Fig. 5), but judging from their almost homonomous wings, the thorax structure of Coxoplectoptera was probably more similar to Permoplectoptera than Ephemeroptera.

***Wing axilla***

The wing articulation in Ephemeroptera, Odonata and Neoptera, and its evolution, is a well discussed topic, closely connected with the evolution of flight [32, 51, 52]. For *M. sharovi* and *M. zalesskyi*, Tshernova [49] does not provide any information on their wing articulation. Very little is known about the wing base in stem-mayflies other than that in [29], [30] and [31]. Kukalová-Peck ([30], Fig. 3) suggests that the wing base in Protereismatidae consists of a series of sclerites delimited by distinct sutures into eight basivenalia and eight fulcalaria. This interpretation was upgraded by the addition of a more proximal series of sclerites, the axalaria and proxalaria (see [29]: Fig. 4). This arrangement of articulary sclerites is consistent with the so called "protowing model", which is recorded in other Paleozoic groups, like the Gerroptera (Odonatoptera) and Palaeodictyopterida. However, the base of the wing of the palaeodictyopteran *Dunbaria quinquefasciata* is not consistent with this model [37]. The wing base is fairly well preserved in all the specimens we studied, but we did not find any convincing evidence that the wing articulation is similar to that proposed by Kukalová-Peck et al. [29]. The structure of wing axilla is rather similar to that in modern mayflies, where the most prominent element of the wing base is the basal plate (BP), attached to the subcosta and radius (Fig. 2A). The proximal margin of the basal plate bears anteriorly a ventral process, which articulates with the pleural wing process (Fig. 2A). In addition, two sclerites, recognizable on the wing base are basalare, located at the anterior base of the wing and subalare, which is a distinct trapezoidal component of the pleura, situated behind PWP. From these elements, PWP can be unambiguously identified on several of the specimens from Tshekarda (Fig. 2B–D) on both the meso- and metathorax. The basal plate is indistinct, but its position can be estimated based on the course of ScP and R veins, which curve around the frontal edge of BP (Fig. 2A–C, arrows). The basalare and subalare were not identified in any specimen of *Misthodotes* from Tshekarda.

Representatives of Neoptera have free second and third axillary sclerites, whereas in Ephemeroptera, the sclerites homologous with the second and third axillary of Neoptera are fused with the basal plate [51], which is why mayflies cannot fold their wings over their abdomen. There is a discussion on whether the non-folding wings in Ephemeroptera is an ancestral condition among Pterygota [28, 31, 55, 56], or a derived state in Ephemeroptera [6, 51, 53], which increases the stability at the wing base when gliding, as suggested by Willkommen and Hörnschemeyer [51]. It is obvious that *M. sharovi* and *M. zalesskyi* could not have folded their wings over their abdomen. If the non-folding wings with the arrangement of axillary sclerites as observed in Ephemeroptera is an apomorphy of mayflies, it is apparently present already in the stem group Permoplectoptera and probably a characteristic of all Panephemeroptera. However, the present observation does not contradict the alternative hypothesis, i.e. fused axillary sclerites and non-folding wings of Ephemeroptera represents a plesiomorphic condition.

***Wings***

From a functional point of view, the general structure of the wings of *M. sharovi* and *M. zalesskyi* differ in several ways from those of recent mayflies. The wings of recent mayflies are used only during mating, oviposition and limited dispersal. Their shape and structure are thus optimized for nuptial and short-range flight [55]. The wings of *Misthodotes* were probably adapted for a greater range of flight activities. As already described by Tshernova [49], the hind wings are only slightly subequal in length compared to the forewings in both *M. sharovi* and *M. zalesskyi*, which corresponds with the size ratio of the meso- and metathorax. Nearly homonomous fore- and hindwings or even slightly broader hindwings occur in all Palaeozoic mayflies [16]. This arrangement is also reported in some Mesozoic lineages such as the families Mesephemeridae and Mickoleitiidae (order Coxoplectoptera), which are a sister group of modern Ephemeroptera [47]. Mayflies with reduced hindwings appeared by the end of the Jurassic, and in most Cretaceous and Cenozoic mayflies hind wings tend to be reduced, while in several taxa they are completely absent [7]. The shape of the wings of *M. sharovi* and *M. zalesskyi* is oval and not enlarged basally, which indicates they were rather slow flyers [55]. Based on the presence of long caudal filaments we assume they were able to glide, especially when descending, as they then act as physical stabilizers [55] and in extant mayflies had an important steering function during gliding in the so called pendular flight [4].

Regarding wing venation, Tshernova [49] provides illustrations of the wing venation of both *Misthodotes* species from Tshekarda; *M. zalesskyi* (Fig. 2 in Tshernova [49]) and *M. sharovi* (Fig. 6 in Tshernova [49]). Carpenter [8] notes discrepancies between Tshernova's figures and her text. In Tshernova [49] the vein CuA is either simple or with a short terminal fork, which is a characteristic of the genus *Misthodotes*. However, in Figs. 2 and 6, Tshernova depicts two long branches of CuA (labelled CuA1 and CuA2), although the bifurcations are drawn as dashed lines. Carpenter [8] concludes (based on a study of Tshernova's drawings, without seeing the actual material), that the vein labelled by Tshernova as CuA2 is probably CuP and the veins CuP1 and CuP2 are anal veins. This interpretation can be corroborated by studying the concavity/convexity of the vein in question, but no information on this topic is provided by Tshernova [49]. Based on our study of the type material, the relief of the wing in that area is flattened and the concavity or convexity of individual veins can not be reliably determined. Given the fact that the branching pattern is very similar to that of *Misthodotes obtusus* Sellards [42] from USA, where there is no doubt about the wing relief and that there is only simple CuA [8], we follow this interpretation for all the species of *Misthodotes* from Tshekarda (Fig. 3).

An important character of the wing venation from a phylogenetic point of view is the costal brace (Figs. 2B, C; 3) as its presence is an apomorphy of Panephemeroptera sensu Sroka et al. [46]. The observed form of the costal brace in *M. sharovi* and *M. zalesskyi* is elongate and distinctly remote from the costal margin, with at least one crossvein connecting the costal brace to the costa, which is a plesiomorphic condition within the mayfly lineage [47]. A similar arrangement occurs in other extinct stem-mayfly taxa and a very basal recent mayfly *Siphluriscus chinensis* Ulmer of the Siphluriscidae (see Fig. 2A).

**Legs**

The legs of *Misthodotes* are not specialized in any particular way so we assume they probably served for walking or clinging to vegetation. In modern mayflies, males have much longer forelegs than females, necessary for grasping a female during copulation, which happens in a specific manner in mayflies, when a male approaches a female from below during swarming. This dimorphism is apparent mainly in the length of foretibia and foretarsus, which are greatly elongated in males [40]. In *M. sharovi* and *M. zalesskyi*, the length of the leg segments can be measured and compared in four specimens (1700/388, 1700/3209, 212/26, 1700/392). Although the foretarsus is longer than hind tarsus, all specimens have very similar foreleg proportions and none of them have more elongated foreleg segments (see Additional file 3: Table S1). Specimen 1700/3209 is a female, 212/26 is possibly also a female, but 1700/388 is probably a male, based on its large eyes (Fig. 1E, F). We therefore assume that the forelegs of males of *Misthodotes* were not similar to those of modern mayflies. Thus, males of *Misthodotes* were probably unable to grasp females in the air during mass swarming and copulation must have taken place most probably after individual encounters on a solid surface. Elongated male forelegs are also not reported in Coxoplectoptera, a sister group of modern Ephemeroptera [47]. We presume the method of copulation in modern mayflies associated with mass swarming is an apomorphic trait of the crown group Ephemeroptera, which is absent in stem groups like Permoplectoptera and Coxoplectoptera. This scenario also corresponds with the structure of the thoracic pleura of *M. sharovi* and *M. zalesskyi* (see above).

Another important leg trait with phylogenetic consequences is the number and arrangement of tarsomeres. Tshernova [49] describe a tarsus with five tarsomeres in *M. sharovi*, with the fifth segment the longest. Carpenter [10] described the tarsus of the related North American species *Misthodotes obtusus* Sellards [42] based on specimens from the collection in the Museum of Comparative Zoology, as relatively short and consisting of four tarsomeres, the middle two being much shorter than the others. The same pattern was reported later in another specimen of *M. obtusus* from the Peabody Museum [8]. The discrepancy in the number of tarsomeres of *M. obtusus* and *M. sharovi* is discussed by Carpenter [8], who considers the placement of *M. sharovi* within *Misthodotes* as doubtful. Based on our observation, the tarsus of *M. sharovi* is composed of four tarsomeres (Fig. 2F) and their arrangement is similar to that in *M. obtusus* (see [8], Fig. 13). This tarsal pattern thus probably represents an apomorphy of Misthodotidae. Closely related Protereismatidae have very long tarsi with 5 tarsomeres, the first tarsomere being the longest and others are subequal [8].

**External genitalia**

Tshernova [49] identified the holotype of *M. sharovi* (1700/3209) as a female and five paratypes as males (212/26, 1700/375, 1700/387, 1700/388, 1700/392). The sex of none of the other specimens of *M. sharovi* (1700/385, 1700/386, 1700/393, 1700/3211, 1700/3212, 1700/3213, 1700/3216) and all the specimens of *M. zalesskyi*, was determined, most likely because the distal part of the abdomen is not preserved.

There is neither a description nor a figure of the genitalia of *M. sharovi* in Tshernova [49], just a statement that the male genitalia in Permoplectoptera are similar to modern mayflies. According to our investigation of the type series of *M. sharovi*, the genitalia are not sufficiently preserved in any specimen for detailed morphological comparisons, although parts of male genitalia are discernible in two specimens, 1700/375— and 1700/392. In both cases, the elongated paired and distally multijointed structures seem to originate from abdominal segment IX, which corresponds with the insertions of male claspers (gonocoxae and gonostyli) and corroborates Tshernova's assignment of these specimens as males (Fig. 4A–D). In modern mayflies, paired claspers are articulated in the same location together with the paired penes [40]. The claspers with elongated gonostyli were present in males of coeval Protereismatidae and also extinct Palaeodictyopterida, which also had a pair of penial lobes [39, 48]. However, specific structures are not identifiable in the *M. sharovi* material.

The absence of an ovipositor in females of *Misthodotes* is also indirect evidence for internalized genitalia comparable to extant relatives and the situation in Protereismatidae, as reported by [48]. Consequently, the oviposition by inserting eggs into plant tissue or similar can be excluded in *Misthodotes*. The eggs were probably laid freely to the water or attached to a substrate.

**Larva**

**Association of larvae with adults of** ***M. sharovi***

Tshernova [49] attributes two larval specimens (1700/374 and 1700/379) to *M. sharovi* based on their proximity and similar body length. Carpenter [8] doubted this association, highlighting the fact that the entire thoracic region, including the wing pads, is missing, judging from Tshernova’s description and figures. Actually, Tshernova [49] illustrated nearly homonomous thoracic wing pads for larval specimen 1700/379, however they are depicted by dotted lines, without further details of the articulation with the thorax and developing venation. Based on our observations, the thoracic region is partially visible in 1700/379, including recognizable fore wing and hind wing pads (Fig. 5A, B, L, M). The hind wing pad overlaps part of tergite III (most probably shifted due to damage of thorax during the process of fossilization). The pattern of lacunae and tracheae on the hind wing pad is poorly preserved. However, the distal ends of radial vein precursors with presumable forks in RP can be discerned (Fig. 5M). The vein precursors are markedly bent distally similar to other Protereismatoidea. Based on preserved remnants of the developing wing pad we assume that the distal ends of the MA and MP vein precursors are located more distally, as occurs in other larvae of Protereismatoidea.

We concur with Tshernova [49] that the larvae of *M. sharovi* have tracheal gills posterolaterally on abdominal segments (Figs. 5A, B; 6A, B). This fact together with the presence of three caudal filaments points to a close relationship with Protereismatidae and Heptabranchia (consisting of Coxoplectoptera and modern mayflies, Ephemeroptera). In Heptabranchia, seven pairs of tracheal gills are present on segments I–VII, with secondary reductions in some recent families [40]. Within Permoplectoptera, nine pairs are present on segments I–IX in Protereismatidae [8, 27]. In *M. sharovi* larvae, gills are also present on segments I–IX, although the basal part of the abdomen is only faintly preserved and the first pair of gills is indistinct. Therefore, the number of gills indicates a close relationship between Misthodotidae and Protereismatidae, with nine pairs possibly being a plesiomorphy of Ephemerida (Permoplectoptera + Heptabranchia).

The shape and proportions of the gills are generally correctly depicted in Tshernova [49], who noticed their lamellar structure is similar to the gills of other Protereismatina [27]. However, the sclerotized ribs present in *M. sharovi* are not recorded in any other Palaeozoic protereismatid larva. Among fossil taxa the presence of gills with more or less developed sclerotized ribs is only recorded for Mesozoic taxa, where it is quite common. Notably, the extinct family Hexagenitidae is characterized by gills with well sclerotized ribs (Fig. 6C). Since Hexagenitidae are undoubtebly crown-group Ephemeroptera, we consider the similarities in the arrangement of gills with that in *M. sharovi* as a convergence possibly resulting from a similar function.

Based on tarsal segmentation the Misthodotidae differ from the Protereismatidae. The larvae of the latter family have tarsi consisting of 5 tarsomeres that terminate in double claws, which is an arrangement identical to that in adults [8], whereas Misthodotidae adults have tarsi with four tarsomeres (see the above description and discussion of adult tarsi). The larva of *M. sharovi* depicted in Tshernova [49] has one-segmented tarsi with a single claw, an arrangement identical to that in Heptabranchia. Based on our observation, *M. sharovi* larvae have tarsi with two claws (Fig. 5G–K), similar to the Protereismatidae. In terms of segmentation the larva of *M. sharovi* has 5 clearly visible tarsomeres similar to the Protereismatidae (Fig. 5G, H).

We conclude that the association of *M. sharovi* adults and larvae can be neither convincingly corroborated, nor refuted. The characters of the larva corresponds with what could have been present in the stem-Ephemeroptera lineage, such as Misthodotidae, but most of these characters are plesiomorphic (tarsi with five tarsomeres, pretarsal double claws, nine pairs of gills, three caudal filaments). The only potentially useful apomorphy of Misthodotidae, i.e. tarsi with four tarsomeres, is not present in the larva. Another fact supporting the association is that all the mayflies in the Tshekarda deposit belong to *Misthodotes* (although the number of species and their delimitation remain doubtful). Thus, it is likely that the taxonomic composition was rather poor and the likelihood of conspecificity high. For these reasons we prefer to keep the species attribution of larva 1700/379 to *M. sharovi* as specified by Tshernova [49]. In the second larva attributed to *M. sharovi* (no. 1700/374), it is more difficult to confirm unequivocally its systematic position because of the poor preservation of the specimen, which lacks wing pads, tarsi and claws. The presence of gills on abdominal segments I–IX indicates a placement within Protereismatoidea sensu Hubbard [21]. The specimen shares several important characters with *M. sharovi* larva 1700/379, such as the number and shape of gills, comparable body size, and presence of specific sclerotized protuberance on posterior margin of terga. Thus, we keep the attribution of the specimen 1700/374 to *M. sharovi*. As discussed above, there are no reliable characters separating adults of *M. sharovi* from *M. zalesskyi*. Consequently, the larva described by Tshernova [49] as *M. sharovi* could have been easily described as *M. zalesskyi* larva. We decided not to change the original attribution mostly to avoid frequent unnecessary changes in the absence of decisive data.

Kluge and Sinitshenkova [24] speculate that the alleged larva of *M. sharovi* belongs to the genus *Phthartus* Handlirsch [19], without providing a detailed reason. Some errors in the early taxonomic accounts of *Pharthus* [17–19] were corrected recently [26] and the genus shares some characters with the larva of *M. sharovi* (plate-like gills, crest on abdominal terga, setation on caudal filaments). However, fossils of *Phthartus* are poorly preserved, with many crucial structures (legs, wing pads, number of gills) not preserved well enough for a detailed comparison. Therefore, we refrain from transferring the larva of *M. sharovi* to *Phthartus*.

**Habitat and life history of larvae**

The larva was undeniably aquatic based on the presence of tracheal gills. Tshernova [50] considers that the absence dense setation on caudal filaments indicates that the larva did not actively swim but had a benthic lifestyle in flowing water. According to our new data, there is setation on the caudal filaments (see Fig. 5P). Nevertheless, this does not exclude a benthic lifestyle, since similar setation is present in recent benthic or even burrowing taxa, which swim only occasionally to either escape from predators or reach the surface of the water for emergence [14, 23].

An important trait overlooked by O. A. Tshernova is the length of the forelegs relative to the other legs. The forelegs are considerably shorter (Fig. 5A, B), which is reminiscent of the larvae of Coxoplectoptera [47]. In our opinion the forelegs were adapted for burrowing in the substrate. This is evidenced by the presence a relatively wide femur, possibly with well sclerotized spines, and a tibia expanded anteriorly, as in many burrowing larvae of Ephemeroidea. The larvae of the closely related Protereismatidae are hypothesized to be predators by ([27], 325), based on the structure of the mandibles of *Kukalova americana* Demoulin [13]. In the description of this larva Kukalová [27] reports that the mandibles are "surprisingly large and broad" and assumes that protereismatid larvae were "in all probability predaceous". However, later Hubbard and Kukalová-Peck [20] claim that these mouthparts are "reminiscent of the mouthparts of some modern generalist feeders". There are figures of this larva in Kukalová [27] in which the mandibles are clearly depicted in the drawing, but not distinct in the photograph. Thus, a reinvestigation of *K. americana* is needed to clarify the arrangement of its mouthparts. Hubbard and Kukalová-Peck [20] review various larvae of Permian mayflies of Protereismatoidea and consider their mouthparts consist of dentate mandibles. The predatory habit was certainly more common in mayfly larvae in the past than nowadays. Of the contemporary mayflies, only 1% are predaceous [3], whereas it was 50% in the Jurassic [50]. As for the larvae of *M. sharovi*, the mouthparts are only faintly discernible and do not have any undeniably predatory modifications.

## Conclusions

The re-examination of the Early Permian material of the genus *Misthodotes* from Tshekarda lagerstätte revealed new morphological traits that increased our understanding of the lifestyle and functional specializations of these representatives of stem mayflies. Notably, we provide the first description of thoracic pleura in stem-Ephemeroptera and identify several plesiomorphies within a mayfly lineage. These include, homonomous meso- and metathorax and lack of fusion and strengthening of the main lateral thoracic sutures associated with the enlargement of dorsoventral flying muscles. We also describe some elements of the wing articulation, such as, the prominent basal plate connected to the pleural wing process in a position similar to that in modern mayflies and the absence of rows of articulatory sclerites. These characteristics, along with the presence of almost homonomous oval wings and long caudal filaments indicate that these insects flew slowly and were capable of both powered flapping and gliding flight.

The adults of *M. sharovi* and *M. zalesskyi* had a protruding orthognathous head with chewing mouthparts indicating they were probably scavengers or herbivores. These Permian species were sexually dimorphic in terms of eye size as in modern mayflies, with males having larger eyes for locating females. However, the males did not have elongated forelegs, which are used by modern taxa to grasp a female in flight during swarming. We hypothesize that the Permian stem group of mayflies did not swarm, since associated morphological traits are absent (enlarged forelegs in males, reduced adult mouthparts connected with short adult lifespan, fusion and strengthening of the pleural suture connected to the enlargement of dorsoventral flying muscles) and are apomorphies of the crown group Ephemeroptera.

We also provide a new interpretation of the tarsal formula of *M. sharovi* and *M. zalesskyi*, which corroborate their affinity with the North American species of *Misthodotes.* The diagnoses of these two species from Tshekarda is problematic due to the great variability within the type series on one hand and the absence of reliable diagnostic characters on the other, therefore, we prefer to retain the original species attribution for the time being.

Association of larvae described as conspecific to *M. sharovi* was evaluated as possible, through not certain, due to the lack of any common apomorphy. Our new observation confirms the relationship of putative larva of *M. sharovi* to the stem-mayfly family Protereismatidae within Permoplectoptera.

## Supplementary Information

**Additional file 1: Fig. S1** Entire slab No. 371 with the positions and numbers of the three fossil specimens of *Misthodotes* marked.

**Additional file 2: Fig. S2.** Forewing length of all specimens of *M. sharovi* and *M. zalesskyi* with a complete preserved forewing.

**Additional file 3: Table S1.** Measurements

## Data Availability

All relevant data are available from the authors. Specimens investigated are accessible from public institutional collections as specified in Methods.

## Abbreviations

A: Anal vein
AEM: Anepimeron
ANp: Anteronotal protuberance
BP: Basal plate
Cl: Claw
Cly: Clypeus
C: Costa
cf: Caudal filaments
Cu: Cubitus
CuA: Cubitus anterior
CuP: Cubitus posterior
Cx: Coxa
Fm, fe: Femur
FS: Furcasternum
KEM: Katepimeron
La: Labrum
LPN: Lateropostnotum
M: Media
MA: Media anterior
MNs: Mesonotal suture
MP: Media posterior
MtNs: Metanotal suture [this term is proposed for the first time]
PCxsA: Anterior paracoxal suture
PCxsP: Posterior paracoxal suture
PLsI: Inferior pleural suture
Pro: Prothorax
PWP: Pleural wing process
R: Radius
RA: Radius anterior
RP: Radius posterior
Sc: Subcosta
ScA: Subcosta anterior (costal brace)
ScP: Subcosta posterior
Ti: Tibia
Tr: Trochanter

## Footnotes

1 The nomenclature of the cephalic and thoracic sclerites, appendages and wing veins used throughout the text is mostly that of Kluge [24] and Willkomenn [52]. The abbreviations are used in alphabetical order.
