# Supplementary figures and images for: Life history, systematics and flight ability of the Early Permian stem-mayflies in the genus Misthodotes Sellards, 1909 (Insecta, Ephemerida, Permoplectoptera)
Source: BMC Ecol Evol. 2021 May 24;21:97. doi: 10.1186/s12862-021-01820-x (PMC8142488; doi:10.1186/s12862-021-01820-x)

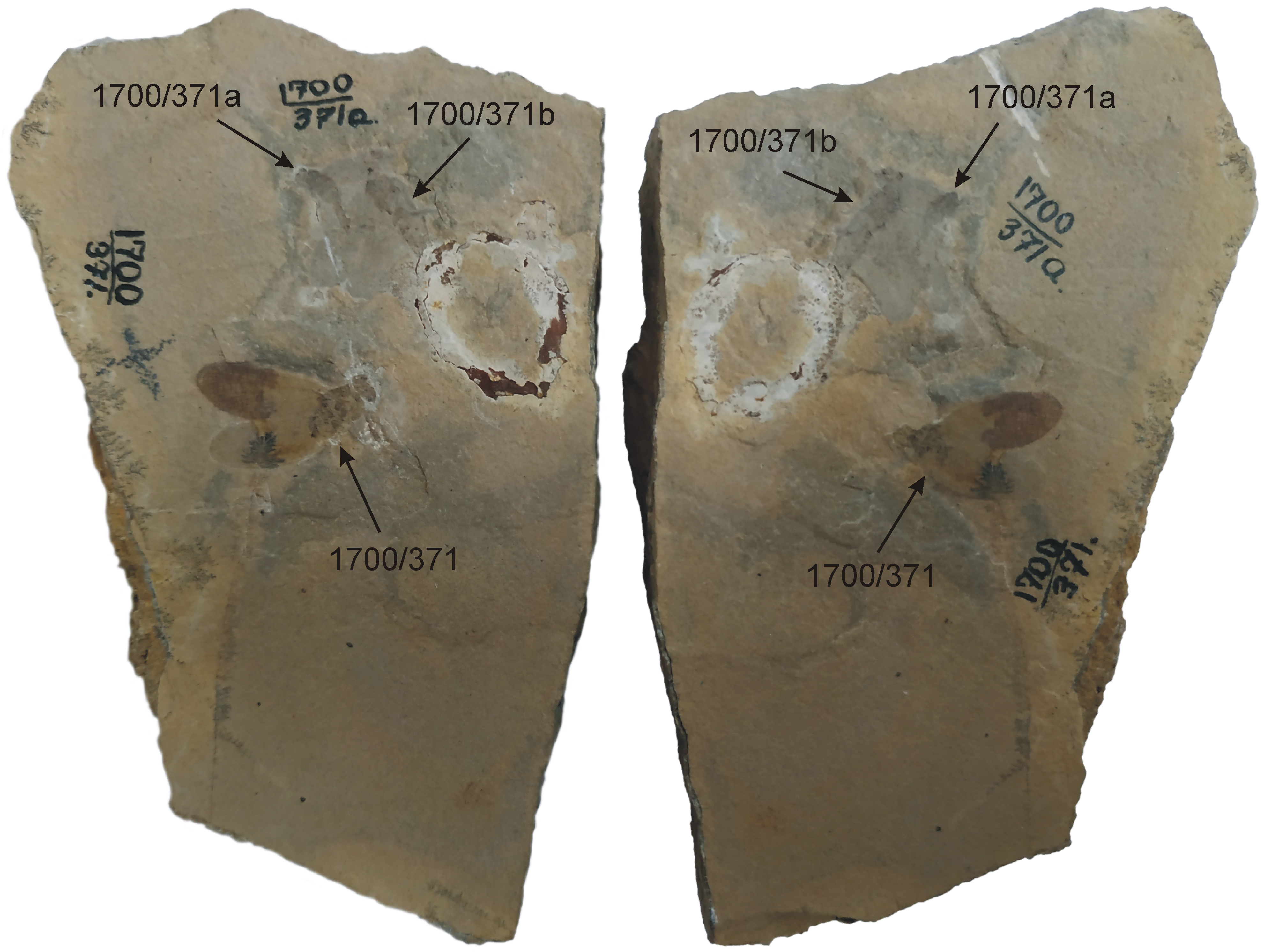

Supplement: Supplementary file 1 — Additional file 1: Fig. S1 Entire slab No. 371 with the positions and numbers of the three fossil specimens of Misthodotes marked. [file 12862_2021_1820_MOESM1_ESM.tif]

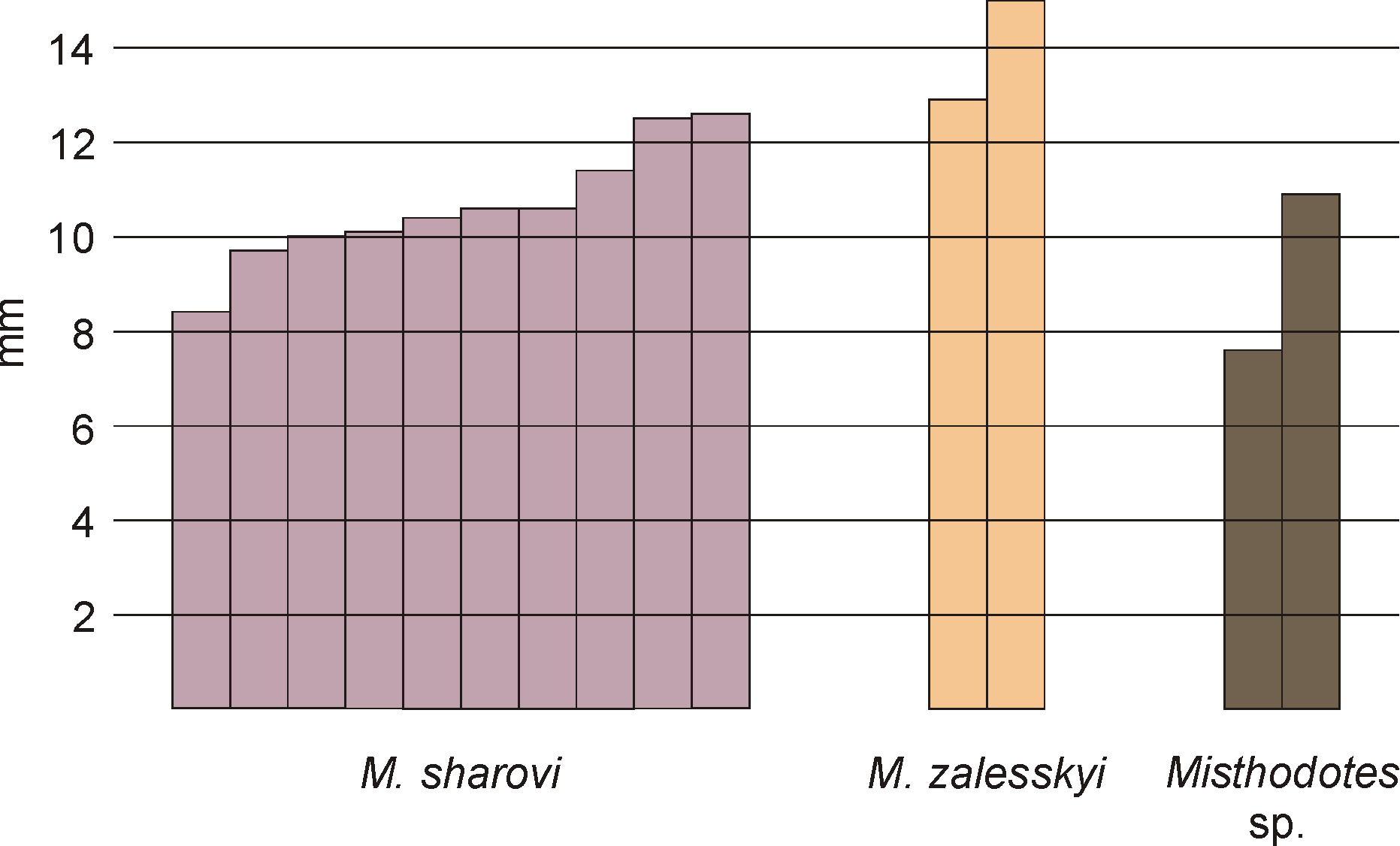

Supplement: Supplementary file 2 — Additional file 2: Fig. S2. Forewing length of all specimens of M. sharovi and M. zalesskyi with a complete preserved forewing. [file 12862_2021_1820_MOESM2_ESM.tif]
