# Supplementary material for: Life history, systematics and flight ability of the Early Permian stem-mayflies in the genus Misthodotes Sellards, 1909 (Insecta, Ephemerida, Permoplectoptera)
Source: BMC Ecol Evol. 2021 May 24;21:97. doi: 10.1186/s12862-021-01820-x (PMC8142488; doi:10.1186/s12862-021-01820-x)
Supplement: Supplementary file 3 — Additional file 3: Table S1. Measurements [file 12862_2021_1820_MOESM3_ESM.doc]

Supplementary Table 1. Measurements

| **Species** | **Specimen No.** | **Body part** | **Measurement (mm)** |
| --- | --- | --- | --- |
| *M. sharovi* | 212/26 | Body length | 15.28 |
|  |  | Forewing length | 12.50 |
|  |  | Hind wing length | 10.50 |
|  |  | Hind wing width | 3.50 |
|  |  | Forefemur length | 1.60 |
|  |  | Foretibia length | 1.21 |
|  |  | Foretarsus length | 2.12; 2.11 |
|  |  | Tarsomere 1 | 0.90; 0.90 |
|  |  | Tarsomere 2 | 0.22; 0.20 |
|  |  | Tarsomere 3 | 0.22; 0.24 |
|  |  | Tarsomere 4 | 0.58; 0.58 |
|  |  | Claw | 0.20; 0.19 |
|  |  | Middle/hind tarsus length^1^ | 2.13; 2.60 |
|  |  | Tarsomere 1 | 2.13; 2.60 |
| *M. sharovi* | 1700/375 | Body length | 12.40 |
|  |  | Forewing length | 10.63 |
|  |  | Forewing width | 3.85 |
|  |  | Hind wing length | 8.50* |
| *M. sharovi* | 1700/385 | Forewing length | 10.63 |
|  |  | Hind wing length | 8.50 |
| *M. sharovi* | 1700/386 | Forewing length | 9.70 |
|  |  | Hind wing length | 8.20 |
| *M. sharovi* | 1700/387 | Body length | 11.11 |
|  |  | Forewing length | 10.10 |
|  |  | Forewing width | 3.38 |
|  |  | Hind wing length | 8.40 |
| *M. sharovi* | 1700/388 | Body length | 12.50 |
|  |  | Ferewing length | 11.38 |
|  |  | Forewing width | 4.25 |
|  |  | Hind wing length | 9.50 |
|  |  | Hind wing width | 3.60 |
|  |  | Forefemur length | 1.30; 1.33 |
|  |  | Foretibia length | 1.25; 1.38 |
|  |  | Foretarsus length | 1.25; 1.29 |
|  |  | Middle/hind femur length^1^ | 2.60; 3.28 |
|  |  | Cerci length | 23.13 |
| *M. sharovi* | 1700/392 | Body length | 9.67 |
|  |  | Forewing length | 8.50; 8.30 |
|  |  | Forewing width | 2.72 |
|  |  | Hind wing length | 7.04; 6.72 |
|  |  | Middle femur length | 1.66 |
|  |  | Middle tibia length | 1.40 |
|  |  | Hind femur length | 2.33 |
|  |  | Hind tibia length | 2.30 |
|  |  | Hind tarsus | 1.38* |
|  |  | Cerci and paracercus length | 12.13* |
| *M. sharovi* | 1700/393 | Forewing length | 10.38 |
|  |  | Forewing width | 3.75 |
| *M. sharovi* | 1700/393a | Forewing length | 10.00* |
|  |  | Forewing width | 3.75 |
|  |  | Hind wing length | 8.88 |
| *M. sharovi* | 1700/3209 | Body length | 15.33 |
|  |  | Forewing length | 12.63 |
|  |  | Forewing width | 4.40 |
|  |  | Hind wing length | 10.63 |
|  |  | Forefemur length | 1.56 |
|  |  | Foretibia length | 1.25 |
|  |  | Foretarsus length | 1.94 |
|  |  | Tarsomere 1 | 0.62 |
|  |  | Tarsomere 2 | 0.24 |
|  |  | Tarsomere 3 | 0.26 |
|  |  | Tarsomere 4 | 0.60 |
|  |  | Claw | 0.22 |
| *M. sharovi* | 1700/3211 | Forewing width | 3.40 |
| *M. zalesskyi* | 1700/371 | Forewing length | 12.88 |
|  |  | Forewing width | 4.90 |
|  |  | Hind wing length | 11.00 |
|  |  | Hind wing width | 4.35 |
|  |  | Middle tibia | 3.36 |
| *M. zalesskyi* | 1700/391 | Forewing length | 15.00 |
|  |  | Forewing width | 5.30 |
|  |  | Hind wing length | 12.63 |
|  |  | Hind wing width | 4.78 |
| *Misthodotes* sp. | 1700/371a | Body length | 7.69 |
|  |  | Forewing length | 7.60 |
|  |  | Hind wing length | 6.06 |
| *Misthodotes* sp. | 1700/371b | Forewing length | 10.94 |
|  |  | Hind wing length | 9.28 |
|  |  | Middle femur length | 2.40 |
|  |  | Hind femur length | 2.28 |
| *M. sharovi* (larva) | 1700/379 | Body length | 14.6* |
|  |  | Head length | 0.96* |
|  |  | Head width | 1.82* |
|  |  | Prothorax length | 1.22 |
|  |  | Prothorax width | 2.43 |
|  |  | Mesothorax length | 1.30* |
|  |  | Mesothorax width | 2.35* |
|  |  | Metathorax length | 1.18* |
|  |  | Forefemur length | 1.04* |
|  |  | Forefemur width | 0.52 |
|  |  | Foretibia length | 1.14 |
|  |  | Foretarsus length (incl. claws) | 1.62 |
|  |  | Tarsomere 1 | 0.26 |
|  |  | Tarsomere 2 | 0.40 |
|  |  | Tarsomere 3 | 0.36 |
|  |  | Tarsomere 4 | 0.22 |
|  |  | Tarsomere 5 | 0.18 |
|  |  | Claw | 0.20 |
|  |  | Middle femur length | 1.95* |
|  |  | Middle tibia length | 1.38 |
|  |  | Middle tarsus length (incl. claws) | 2.32* |
|  |  | Tarsomere 1 | 0.36 |
|  |  | Tarsomere 2 | 0.36 |
|  |  | Tarsomere 3 | 0.30 |
|  |  | Tarsomere 4 | 0.26 |
|  |  | Tarsomere 5 | 0.26 |
|  |  | Claw | 0.78* |
|  |  | Hind femur length | 2.28* |
|  |  | Hind tibia length | 1.06* |
|  |  | Hind tarsus length (incl. claws) | 1.50* |
|  |  | Tarsomere 1 | 0.26 |
|  |  | Tarsomere 2 | 0.18 |
|  |  | Tarsomere 3 | 0.16 |
|  |  | Tarsomere 4 | 0.19 |
|  |  | Tarsomere 5 | 0.18* |
|  |  | Claw | 0.53* |
|  |  | Abdomen length | 9.94* |
|  |  | Cerci length (right, left) | 3.94*; 1.82* |
|  |  | Paracercus length | 2.90* |
| *M. sharovi* (larva) | 1700/374 | Body length | 14.60 |
|  |  | Cerci length (right, left) | 6.82*; 6.14* |
|  |  | Paracercus length | 6.44* |

Remarks: Where both right and left side of the paired structures are measurable, both values are listed. Where the same structure is completely visible on + and - imprint of the same specimen, it is measured separately and mean value is listed.

*Incomplete

^1^Middle and hind legs not unambiguously identifiable
